# Reclassifying Hepatic Cell Death during Liver Damage: Ferroptosis—A Novel Form of Non-Apoptotic Cell Death?

**DOI:** 10.3390/ijms21051651

**Published:** 2020-02-28

**Authors:** Ricardo U. Macías-Rodríguez, María Eugenia Inzaugarat, Astrid Ruiz-Margáin, Leonard J. Nelson, Christian Trautwein, Francisco Javier Cubero

**Affiliations:** 1 Department of Gastroenterology, Instituto Nacional de Ciencias Médicas y Nutrición Salvador Zubirán, Mexico City 14080, Mexico; ruizm.astrid@gmail.com; 2 Department of Internal Medicine III, University Hospital RWTH Aachen, 52074 Aachen, Germany; m.euge.inzaug@gmail.com (M.E.I.); ctrautwein@ukaachen.de (C.T.); 3 MICTLÁN-Network (Mechanisms of Liver Injury, Cell Death and Translational Nutrition in Liver Diseases Research Network), Mexico City 14080, Mexico; 4 Liver Fibrosis and Nutrition Lab (LFN Lab), Mexico City 14080, Mexico; 5 Institute for Bioengineering (IBioE), School of Engineering, Faraday Building, The University of Edinburgh, Edinburgh EH9 3 JL, UK; l.nelson@ed.ac.uk; 6 Department of Immunology, Ophthalmology & ENT, Complutense University School of Medicine, 28040 Madrid, Spain; 7 12 de Octubre Health Research Institute (imas12), 28041 Madrid, Spain

**Keywords:** ferroptosis, liver disease, liver fibrosis, cell death, lipid peroxidation

## Abstract

Ferroptosis has emerged as a new type of cell death in different pathological conditions, including neurological and kidney diseases and, especially, in different types of cancer. The hallmark of this regulated cell death is the presence of iron-driven lipid peroxidation; the activation of key genes related to this process such as glutathione peroxidase-4 (*gpx4*), acyl-CoA synthetase long-chain family member-4 (*acsl4*), carbonyl reductase [NADPH] 3 (*cbr3*), and prostaglandin peroxidase synthase-2 (*ptgs2*); and morphological changes including shrunken and electron-dense mitochondria. Iron overload in the liver has long been recognized as both a major trigger of liver damage in different diseases, and it is also associated with liver fibrosis. New evidence suggests that ferroptosis might be a novel type of non-apoptotic cell death in several liver diseases including non-alcoholic steatohepatitis (NASH), alcoholic liver disease (ALD), drug-induced liver injury (DILI), viral hepatitis, and hemochromatosis. The interaction between iron-related lipid peroxidation, cellular stress signals, and antioxidant systems plays a pivotal role in the development of this novel type of cell death. In addition, integrated responses from lipidic mediators together with free iron from iron-containing enzymes are essential to understanding this process. The presence of ferroptosis and the exact mechanisms leading to this non-apoptotic type of cell death in the liver remain scarcely elucidated. Recognizing ferroptosis as a novel type of cell death in the liver could lead to the understanding of the complex interaction between different types of cell death, their role in progression of liver fibrosis, the development of new biomarkers, as well as the use of modulators of ferroptosis, allowing improved theranostic approaches in the clinic.

## 1. Introduction

In the liver, the presence of hepatocyte death is reflected in the levels of serum transaminases, which are the most widely used markers of hepatic function [1]. Moreover, these markers have prognostic value in a wide range of chronic liver diseases, often involving persistent inflammation of any underlying cause, such as hepatitis C virus infection (HCV), alcoholic liver disease (ALD), non-alcoholic steatohepatitis (NASH), drug-induced liver injury (DILI), and hepatocellular carcinoma (HCC) [2,3,4].

Cell death represents not only the endpoint in response to a variety of insults but can also be self-executed in a process called programmed cell death (PCD). Distinct forms of hepatocyte cell death include apoptosis (a typical form of PCD), necrosis, pyroptosis, necroptosis, and autophagy. The differences between these modes of liver cell death include distinct changes in the nucleus, cytoplasm, and other organelles such as lysosomes. Despite having different pathways involved, all these mechanisms result in irreparable cellular dysfunction, leading to cell death [5,6,7,8,9].

Very recently, a new type of cell death termed ferroptosis, in which the hallmark is the contribution of iron to the development of oxidative cell damage, has been described [10]. Studies on ferroptosis have been done mainly in animal models of cancer as well as renal and neurological injury [11,12,13,14,15]. Current studies suggests a possible association between ferroptosis and different types of chronic liver disease including hemochromatosis, ALD, HCV, NASH, and HCC, as well as DILI. An imbalance in iron metabolism as well as reactive oxygen species (ROS)-induced lipid peroxidation has been recognized as a mechanism of liver injury in these diseases [16,17,18].

In the first part of this review, we give a brief overview of the common types of cell death, which highlights some fundamental differences with ferroptosis; next, we discuss general mechanisms in ferroptosis and evidence indirectly involving ferroptosis and iron-mediated cellular damage in liver diseases. Finally, the clinical implications of recognizing this type of cell death are described.

## 2. General Mechanisms of Liver Cell Death

Clinical data and experimental models clearly suggest that different types of hepatocyte death trigger the progression of liver disease from different etiologies. The prevailing type of cell death is tissue, spatiotemporal, and situation-specific, and it seems to be a constitutive mechanism in the normal development and physiology of a tissue [19]. Recognizing the specific type of cell death in liver disease is crucial for the detection of specific risk factors involved in the progression and perpetuation of the damage. In addition, the understanding of the mode of cell death will help to develop the novel targeted therapies, the dissection of molecular mechanisms, and the interactions between different pathways involved in hepatocyte damage [6]. Table 1 shows the main characteristics of each type of cell death.

## 3. Apoptosis

Apoptosis, a highly regulated cell death and one of the most studied, has been detected in many experimental models of liver disease [35,36]. Shrunken cells, membrane blebbing, nuclear condensation, and fragmentation are the main morphological features of this type of cell death [9]. These changes finally lead to the breakdown of the cell into small fragments called apoptotic bodies. Kupffer cells (liver resident macrophages that are part of the reticuloendothelial system (RES)) then engulf these apoptotic bodies causing the enhanced expression of death ligands (TNF, TNF-related apoptosis-inducing ligand (TRAIL), and Fas ligand (FasL), eliciting further immunological responses, perpetuating and causing secondary damage [37,38]. Another hallmark of apoptosis is the contribution of specific caspases that are involved in the initiation (caspase 2, 8, 9 and 10) and execution (caspase 3, 6 and 7) of cell death [20].

Apoptosis can be categorized as intrinsic or extrinsic, depending on whether the initial signal is due to microenvironmental perturbations such as DNA damage and ROS overload or endoplasmic reticulum (ER) stress (intrinsic), or elicit by extracellular microenvironment alterations and mediated by death receptors (DRs) (extrinsic) [21]. The intrinsic pathway initially involves the participation of Bcl-2 family members and both the release of cytochrome C and caspase activation. After noxious stimuli are released (e.g., ROS) from different sources, members of the Bcl-2 family of proteins are differentially regulated, leading to mitochondrial outer membrane permeabilization. This means that a highly regulated interaction between pro- and anti-apoptotic signaling occurring in members of this family is responsible for the control of the mitochondrial pathway in apoptosis [39]. This interaction takes place on the outer membrane of mitochondria and includes three different subgroups within the Bcl-2 family: BH3-only proteins (initiates apoptosis, includes *Bid*, *Bim,* and *Puma*), pro-survival elements (such as Bcl-2), and the effectors of apoptosis (*Bax* and *Bak*) [40]. When the balance between the different proteins from the three groups favors apoptosis, there is a reduction in the energy metabolism in mitochondria caused by a derangement of the electron transport chain and the release of proteins that activate caspases and trigger the alteration of the redox potential [41,42]. Once the interaction between the Bcl-2 proteins takes place, the next event involves permeabilization of the mitochondrial outer membrane, allowing the release of intermembrane space proteins, including cytochrome C to the cytosol [43]. Then, cytochrome c binds to APAF1 (apoptotic protease-activating factor-I) and in the presence of dATP enables apoptosome formation [44]. The apoptosome allows the activation of pro-caspase-9 to caspase-9, which in turn activates the effector caspases 3 and 7 [45,46].

The extrinsic pathway, on the other hand, initiates with the binding of an extracellular death ligand (TNF, Fas ligand, or TRAIL) onto the surface of the extracellular domain of a transmembrane DRs, such as Fas cell surface death receptor (Fas) or TNF receptor superfamily member 1A (TNFR1) [46,47]. DRs recruit the Fas-associated death-domain (FADD) and translate the signal into the cytoplasm, leading to the assembly of the death-inducing signaling complex (DISC). This complex is formed by interactions involving Fas receptor, leading to the recruitment of FADD (Fas-associated death domain protein) and finally caspase-8 (and caspase-10), triggering the dimerization and activation of these caspases [44,46,48,49]. The binding of TNF to TNFR1 enables the formation of a complex that includes receptor-interacting serine/threonine-protein kinase 1 (RIPK1), FADD, and caspase-8, initiating apoptosis (in the absence of FLIPs, or FLICE-inhibitory proteins) [49,50].

## 4. Necrosis and Necroptosis

Necrosis was largely known as a “chaotic” response to different stressors, including physicochemical stress, and it is characterized by increased cytoplasmic granularity, mitochondrial damage, impairment in the production of energy (ATP), and the subsequent failure of ATP-dependent ion pumps. The final result is an acute “osmotic” change (oncosis) in the cell and cell organelles, leading to swelling and eventually rupture of the membrane, with the release of intracellular content (including damage-associated molecular patterns (DAMPs) and, finally, eliciting a strong inflammatory/immune response [6,9,22]. 

Although necrosis was initially regarded as an essentially ‘disorganized’ cellular response, it has now been shown that there are specific pathways that regulate necroptosis, including a *necrosis-like* mode of regulated cell death [22]. Necroptosis is induced when extracellular or intracellular stimuli are detected by TNFR1, Fas, or Toll-like receptors (TLR)-3 and 4, and it includes the participation of different elements such as receptor-interacting serine/threonine protein kinase 1 (RIPK1), RIPK3, and mixed lineage kinase domain-like (MLKL) [22,51]. It has been demonstrated that necroptosis is involved in modulating adaptive immunological functions, such as maintaining T-cell homeostasis in adults [52].

There are several factors that can initiate necroptosis, including TNF, Fas, TRAIL, IFN, LPS, dsRNA, DNA damage, endoplasmic reticulum (ER) stress, viral infection, and anticancer drugs [22]. Following TNF stimulation, TRADD and RIPK1 are recruited to the TNF receptor, forming the complex I. Here, RIPK1 is ubiquitylated by cIAPs (Lys63-linked) or LUBAC (linear ubiquitylation), stabilizing the complex and recruiting nuclear factor (NF)-kB signaling pathway complexes. Further stimulation and the action of specific enzymes results in the deubiquitylation of the complex and forms the complex II composed of oligomerized FADD, TRADD, and RIPK1. This complex recruits and activates caspase-8, finally leading to apoptosis. However, when caspase-8 activity is not available, deubiquitylated RIPK1 recruits RIPK3 via RHIM (RIP Homotypic Interaction Motif) interaction, undergoing autophosphorylation and necrosome formation. In this complex, RIPK3 recruits and phosphorylates MLKL, forming active oligomers that finally translocate to and destabilize the plasma membrane through interaction with phosphatidylinositide (PI) [51]. This causes cell membrane permeabilization and cellular death, and it is characterized by oncosis, swelling of the organelles, and nearly no change in the nuclei until later stages when chromatin condensation is observed [23,53]. 

## 5. Autophagy

The main function of autophagy is to contribute to cellular renewal, allowing the lysosomal degradation of different components, including extracellular material and membrane proteins as well as cytosolic components and organelles [28]. In autophagy, cytoplasmic materials are delivered to the lysosome, the autophagosomes are formed from autophagy-related (ATGs) proteins, and finally, the contained elements are degraded. Three types of autophagy have been described, including (a) Macroautophagy, (b) Microautophagy, and (c) Chaperone-mediated autophagy. Canonical macroautophagy incorporates cytoplasmic components into lysosomes and is the best described type of autophagy [29]. In this section, the term autophagy refers to macroautophagy. 

Several stimuli lead to the induction of autophagy, including starvation, drugs (e.g., rapamycin, amiodarone, loperamide) and some diseases [30,31,54]. Autophagy has different stages, including (a) Initiation of autophagosome formation, (b) Elongation, (c) Maturation, and (d) Fusion with lysosomes [9]. In the first step of autophagy, an isolation membrane (phagophore) is usually formed around a small part of the cytoplasm, invasive microbes, or an organelle; then, it is sequestered by a membrane-sac structure that is later elongated, leading to the formation of a double-membrane vesicle: the autophagosome. The formation of the autophagosomes initiates with the presence of metabolic stressors and depends on the coordinated action of the ATGs proteins. Then, the autophagosome matures and sequesters completely the intracellular cargo (its outer membrane fusing with the lysosome), forming an autolysosome, where its inner membrane and content are degraded by the acid hydrolases [28,31,55]. The resulting macromolecules diffuse to the cytoplasm through membrane permeases [56] where they are used for metabolic recycling. 

Specifically, in cell death, autophagy can have different roles: (a) autophagy-associated cell death; (b) autophagy-mediated cell death, and (c) autophagy-dependent cell death [32]. In the first two, autophagy has a secondary role, depending on the presence of other types of cell death (e.g., apoptosis), which are responsible for executing cell death itself. In contrast, autophagy-dependent cell death does not require other types of cell death. Interestingly, autophagy seems to act as a cell death backup mechanism, being activated when apoptosis is inhibited. In Bax/Bak double knockout mice—which are resistant to apoptosis—the pathways and morphological changes indicate the activation of autophagy when cells are exposed to death ligands [57]. 

Autophagy plays an important role in the regulation of metabolism in the liver, energy production, and as a quality control checkpoint of organelles such as mitochondria. The disruption of this pathway has been linked to various liver diseases including NAFLD, HCC, and chronic viral hepatitis, among others, [29] and although autophagy has been mainly described as a “recycling” mechanism, there is evidence showing that autophagy could be associated to liver cell death. In a study of 12 patients with acute liver failure (ALF) secondary to anorexia nervosa, liver biopsies showed the formation of autophagosomes in electron microscopy, as well as changes in immunostaining showing expression of ATG5 in controls and patients, and evidence of endoplasmic reticulum (ER) stress only in the patient group; the findings in the liver biopsy reasonably excluded apoptosis or necrosis as the predominant mechanism of liver injury. Although a more detailed analysis of the mechanisms of cell death would be recommended, the findings in this study suggest that autophagy could elicit cell death under some specific circumstances [24].

Finally, there is evidence showing a link between the activation of autophagy and the development of ferroptosis through a process known as “ferritinophagy”, featuring the autophagic degradation of ferritin. In this process, the nuclear receptor coactivator 4 (NCOA4, a selective cargo receptor for the turnover of ferritin) helps to maintain iron homeostasis, contributing to ferritin degradation, thus increasing iron levels and promoting the development of ferroptosis. Autophagy promotes ferroptosis by the degradation of ferritin [58,59]. 

## 6. Pyroptosis

Pyroptosis is a type of regulated cell death that it is mainly involved in proinflammatory events. This means that while the other types of regulated cell death can be observed in normal physiological processes, such as embryogenesis, pyroptosis is present always as a non-physiologic response to several extracellular stimuli (e.g., TNF, IFN, and TLR ligands) and to different intracellular pathogens [25,26]. Initially, pyroptosis was described as being dependent on caspase 1 activation; however, recent findings show that it can be triggered by other caspases (such as caspase-3), whilst it can also be dependent on pore formation by the gasdermin (GSDM) protein family [27].

In order to induce canonical pyroptosis mediated by inflammasomes, two steps are required: (a) a priming step, where mediators are transcriptionally generated; and (b) an inflammasome activation/assembly phase [25].

In the first step, the cell is “primed” by extracellular ligands such as TNF and pathogen-associated molecular patterns (PAMPs), resulting in the enhanced gene expression of non-active or immature forms of different signaling proteins, including pro-*IL1ß*, pro-*IL18*, and gasdermin D (GSDMD). In the second step, DAMPs and components of intracellular pathogens bind to pattern recognition receptors (PRRs), which can include nucleotide-binding domain-like receptors (NLR) pyrin and HIN domain (PYHIN) or tripartite motif (TRIM) families. This allows inflammasome assembly and the activation of caspase-1, which further cleaves pro-ILs into their active forms (IL-1*ß* and IL-18). Moreover, active caspase-1 promotes the proteolytic cleavage of GSDMD, promoting the release of the N-terminal domain of GSDMD, which translocates to the plasma membrane, undergoes oligomerization, and generates membrane pores. In contrast, the non-canonical inflammasome involves the activation of caspase-4 or 5 (in humans), or caspase-11 (in mice), by intracellular LPS. Activated caspases cleave some lesser-known targets, including GSDMD, which then, as in the canonical mechanism, translocate to the membrane, leading to pore formation [26]. 

## 7. Iron Metabolism

The metabolism of iron is tightly regulated by different molecules and transporters. However, although a specific mechanism responsible for the direct elimination of iron has not been elucidated, modulation in the absorption of dietary iron occurs depending on the iron stores in the body and other conditions such as inflammation and hypoxia. This modulation is achieved through a delicate interplay involving the RES–gut–liver axis [60].

Dietary iron is taken up by intestinal epithelial cells (IECs) through the luminal membrane, internalized, stored, and finally released to the circulation via ferroportin. In the apical membrane of enterocytes, ferric iron (Fe^3+^) is reduced to its ferrous state (Fe^2+^) by duodenal cytochrome b (D-cytb), and then, it is internalized into the enterocytes by divalent metal-ion transporter 1 (DMT1). Then, iron is stored as ferritin or distributed to target cells/organs via the circulation either through ferroportin and/or bound to transferrin (and to a lesser extent, other low-molecular-weight compounds e.g., citrate) [60,61]. Finally, iron is taken up by cells via the surface transferrin receptor (TfR1). The non-transferrin-bound iron (NTBI) is responsible for the oxidant-mediated cellular injury, and its levels increase with transferrin saturation. In physiological conditions, transferrin is saturated 30% with iron, while a value <16% indicates iron deficiency and >45% reflects iron overload; when the saturation is higher than 60%, the risk of iron accumulation in different cells increases [61].

One of the most important molecules regulating iron balance is hepcidin, which is produced in the liver and secreted into the circulation, playing a key role in iron homeostasis. Hepcidin modulates iron efflux into the plasma by altering the function and inducing the degradation of the ferroportin present in macrophages and enterocytes [62]. The expression of hepcidin is controlled through the bone morphogenetic protein (BMP) and JAK2/STAT3 signaling pathways, which, can be influenced by inflammation [63,64]. Responses to iron levels/hypoxia can be explained by a systemic and a compartmentalized effect, the latter referring to a local effect in enterocytes or macrophages, where the above-mentioned pathways are involved. 

Iron is a critical growth factor for several pathogens (including in tuberculosis and malaria); therefore, iron levels are carefully controlled in the body by protein chaperones such as transferrin and ferritin. So, reducing levels of iron during inflammation would naturally contribute to limiting its availability in order to limit pathogen proliferation [65]. Interestingly, cytoplasmic-soluble free iron is an important source for oxidation reactions that produces hydroxyl and peroxyl radicals that, in turn, contribute to the peroxidation of PUFA-PLs [66]. As a consequence, cells with an excess of iron are more sensitive to ferroptosis [10].

## 8. Ferroptosis

First described as a form of cell death in cancer by Stockwell and colleagues [10], the key event of ferroptosis is the iron-driven production of ROS, in which the iron possibly originates both from intracellular organelles as well as cytoplasm iron stores and iron-containing enzymes. Morphologically, ferroptosis is characterized by shrunken, electron-dense mitochondria, rupture of the outer mitochondrial membrane, and the presence of lipid peroxidation [10,33,67,68]. Currently, ferroptosis can be detected by measuring lipid peroxidation (LPO), increased PTGS2 expression (genetic and protein), and decreased content of the reduced form of nicotinamide adenine dinucleotide phosphate (NADPH) [66,69,70]. 

**Figure 1:**
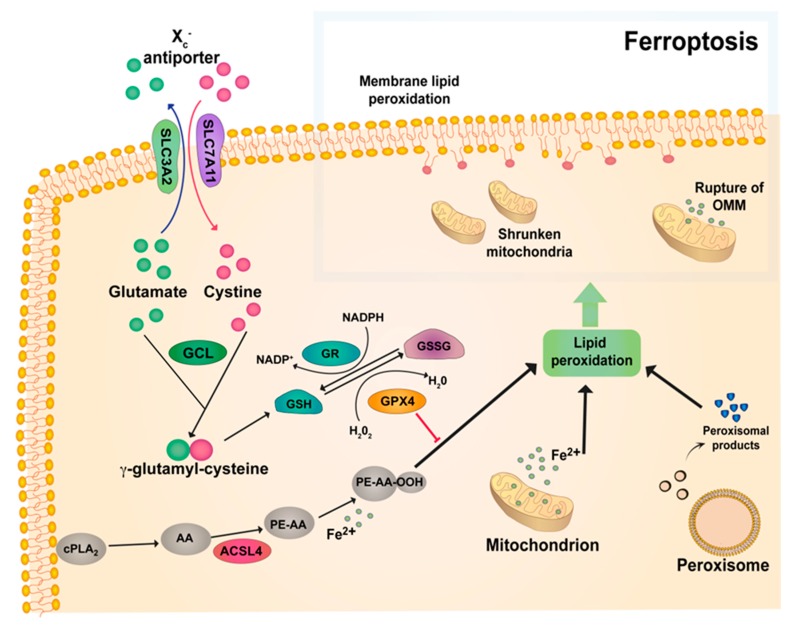
General mechanisms of ferroptosis. The Xc^-^ antiporter system consisting of the SLC7A11 and SLC3A2 subunits, which allows the extrusion and internalization of glutamate and cysteine. γ-glutamylcysteine ligase (GCL) binds glutamate and cysteine together to form γ-glutamylcysteine. Later, glutathione (GSH) is formed, which is used then by glutathione peroxidase (GPX4) to scavenge ROS and lipid reactive species produced by peroxisomes and the release of iron from mitochondrion. Glutathione reductase (GR) catalyzes the reduction of glutathione disulfide (GSSG) to GSH using nicotinamide adenine dinucleotide phosphate (NADPH). ACSL4 facilitates the formation of phospholipids (PL), and finally oxidized-PE interacts with Fe^2+^, triggering iron-dependent lipid peroxidation. Downregulation of the GPX4 cycle leads to lipid peroxidation, which causes the breakthrough of lipid membranes, disruption of the mitochondrial electron transport chain, and shrunken mitochondria. PE, phosphatidylethanolamine; AA, arachidonic acid; ACSL4, long-chain-fatty-acid-CoA ligase 4.

The cystine/glutamate antiporter Xc^-^ plays a crucial role in ferroptosis. The Xc^-^ system consists of the SLC7A11 and SLC3A2 subunits, allowing the extrusion and internalization of glutamate and cysteine, respectively [71]. This allows for the ATP-dependent peptide coupling of cysteine and glutamate to form y-glutamylcysteine (GGC), which is catalyzed by y-glutamylcysteine ligase (GCL). Glutathione synthetase (GSS) joins GGC to glycine to produce glutathione (GSH). Finally, GSH is utilized by glutathione peroxidase (GPX) to scavenge ROS and lipid reactive species produced by the disruption of lipid membranes, the mitochondrial electron transport chain and possibly from the release of iron from iron-containing enzymes [34,72,73] (Figure 1).

There are several isozymes of GPX (GPX1–GPX8) [74], of which GPX4 is the most important in protecting against lipid peroxidation driven by ferroptosis. Although the downstream pathway of GPX4 is not well understood, it has been demonstrated that several factors (e.g., erastin) can increase ferroptosis by indirectly downregulating the GPX4 cycle. Interestingly, cyclooxygenase-2 (COX-2; see below) has been identified as a marker of ferroptosis (PTGS2 gene) together with other markers, including changes in NADPH levels and lipid peroxidation [70,75]. 

There is evidence showing ferroptosis-induced endoplasmic reticulum stress after the pharmacological inhibition of cystine-glutamate exchange [76]. Upon ER stress and pharmacological-induced ferroptosis (v.gr. with erastin and sorafenib), there is an increased expression of PUMA through the ER stress–mediated PERK–eIF2a–ATF4–CHOP pathway, but without inducing apoptosis, suggesting a link between apoptosis and ferroptosis [77]. The precise role of ER stress and ferroptosis needs to be further assessed.

### 8.1. The Role of Lipids in Ferroptosis

There is evidence suggesting that lipids are involved as mediators of ferroptosis orchestrating its final steps, both as small lipid particles interfering with the function of the membranes and membrane proteins, and also as lipids eliciting intracellular signaling and stimulating other pathways involved in cell death [10,33]. In fact, ferroptosis is sometimes described as death by LPO due to their tight association [66]. 

Lipids and its metabolites are mediators of many biological responses [78,79]. The role of lipids exerting different functions in inflammation, immunology, metabolism, and as a component of membranes has been extensively studied. One of the most important features is their function as effectors regulating growth-related signals, gene expression, and cell survival [78,80]. The equilibrium between cell proliferation and cell death mediated by lipids is maintained by an intricate network that includes many different enzymes. These proteins are readily available to catalyze lipids derived from intracellular and extracellular sources, yielding metabolites derived mainly from arachidonic acid (AA) and other fatty acids, including prostaglandins, leukotrienes, and lipoxins [81,82,83]. The action of the different lipids depends on the lipid itself, their specific receptors, and the cell tissue type. For example, in decompensated cirrhosis, prostaglandin E2 (PGE2) mediates immunosuppression [84]. 

In the context of ferroptosis, the oxidation of polyunsaturated-fatty-acid-containing phospholipids (PUFA-PL), which occurs not only in the plasma membrane but also in other subcellular locations, seems to play a central role [10,33]. As a result of the LPO of polyunsaturated fatty acids (PUFA), a wide range of oxidation products are produced, such as malondialdehyde (MDA) and 4-hydroxynonenal (4-HNE), which can modulate transcription factors and induce cell death [67]. Moreover, among the several enzymes described to drive ferroptotic cell death, lipoxygenases (LOX) have been found to be the most important, [33,68] even although the precise mechanism is still not fully understood. However, recent studies show that ferroptosis inhibitors, including LOX inhibitors, execute an antioxidant function preventing the autooxidation and non-enzymatic destruction of membrane PUFA-PL [69]. Finally, evidence indicates that upon the induction of ferroptosis, COX-2 overexpression is induced. Since its inhibition through indomethacin did not show changes in ferroptotic cell death, COX-2 seems to be only a marker of ferroptosis, but it does not seem to play a key role in this process [12]. This further contributes to add more complexity to the exact role of lipids and its by-products in ferroptosis.

### 8.2. Keap1-Nrf2 System

The Keap1-Nrf2 system plays an important role as a sensor of oxidative stress/cellular damage, regulating the expression of genes related to detoxifying and/or antioxidant enzymes [85,86]. Keap-1 (Kelch-like erythroid cell-derived protein with CNC homology (ECH)-associated protein 1) is a sensor of cell damage, including reactive oxygen species (ROS) and electrophiles. These stress signals induce Nrf2 (NF-E2-related factor 2) activation, which in turn activates the expression of several genes involved in the antioxidant response. Keap1 is found mainly on the perinuclear cytoplasm, where it is attached to the actin cytoskeleton. Disruption of the cytoskeleton allows the release of Nrf2 from actin-bound Keap1, and thus the translocation and nuclear entry of Nrf2 [87,88]. In the nucleus, Nrf2 forms a heterodimer with small MAF (sMAF), and then this heterodimer activates the gene expression of detoxifying enzymes through its binding to the antioxidant response elements (AREs)/electrophile response elements (EpREs). The result is the activation of multiple defense enzymatic systems, leading to cytoprotective processes aimed at preserving the integrity of the cell and its components [89,90]. 

The basic structural components of the Keap1-Nrf2 system include a trimer, consisting of one Nrf2 and two keap1 molecules. Importantly, there are multiple cysteine residues on keap1, which react according to the type of electrophile [91]. For example, Cys151, Cys273, and Cys288 are cysteine residues acting independently or collaboratively as sensors of oxidative stress. On the other hand, the oxidative changes of Keap1 also cause modifications, leading to its inactivation and finally to Nrf2 stabilization and nuclear accumulation [92,93].

Under normal conditions, in the absence of oxidative stress, Nrf2 undergoes degradation via the ubiquitin–proteasomal pathway; therefore, its levels are very low [87,93]. This process is mediated by Keap1, which is an E3 ubiquitin ligase substrate-recognition subunit targeting Nrf2, and it is therefore ubiquitinated by the Keap1-Cul3 E3 ligase and degraded. Upon exposure to ROS and other molecules, levels of Nrf2 increase considerably due to the inability of Keap1 to ubiquitinate Nrf2, promoting Nrf2 accumulation in the nucleus and inducing nuclear target genes associated with antioxidant, metabolic, and detoxifying enzymes [86].

Additionally, Nrf2 may play an important role as an anti-inflammatory factor given that the Nrf2 gene binds to the promoter region of some pro-inflammatory genes, blocking the transcription of lipopolysaccharide-induced cytokines such as *IL-1β* and *IL-6* [94].

Of interest, cysteine residues Cys273 and Cys288 on Keap1 can also react with 15-deoxy-D12,14 prostaglandin J2 (15d-PGJ2), thus modulating its function and exerting some of the effects related to this prostaglandin. Moreover, 15d-PGJ2 activates p53 expression via Nrf2 upregulation of heme oxygenase-1 (HO-1), possibly increasing the production of iron. Consequently, activation of the Keap1–Nrf2 system could play an important role in the final step of ferroptosis, particularly in the interaction between iron, lipid mediators, and ROS [95,96]. 

Finally, there is data showing that Nrf2 induces some of the ferroptosis-related genes, such as glutathione peroxidases (GPXs), suggesting an intricate interaction between different systems and the participation of different cellular levels in this type of cell death. Supporting this, the Keap1–Nrf2 system has been also implicated in the regulation of the heme metabolism, including iron trafficking, erythrocyte survival, erythropoiesis, which has been extensively addressed, as discussed in a recent review [97,98]. Furthermore, Nrf2 can induce ferritin, modulate the expression of ferroportin (fpn1), and enable iron to incorporate into pirin (PIR), which is a nuclear non-heme iron-binding protein that regulates the NF-kB signaling pathway. Overall, the coordinated action of the Keap1–Nrf2 system depends upon the prevailing redox cellular state, together with the available iron [99].

### 8.3. Interaction between Iron and Oxidative Stress 

Two types of iron can be found in the body: free iron and bound iron. The type related to oxidative stress is free iron, owing to its instability and high reactivity. Iron is involved in the Fenton reaction, where hydrogen peroxide (H_2_O_2_) is catalyzed by iron, yielding the highly reactive hydroxyl radical:

Fe^2+^ + H_2_O_2_ → Fe^3+^ + HO^.^ + OH^-^

The high amount of iron distributed throughout the body, together with the constant mitochondrial production of H_2_O_2_, renders this reaction an important source of free radicals, [100] leading to oxidative damage to lipids, proteins, and DNA.

In addition to the interplay between iron and ROS, some reactive nitrogen species (RNS) can react with the iron in some proteins, causing dysfunction, such as cellular toxicity, metabolic enzyme damage, or permeability transition pore stimulation, that ultimately can lead to cell death [101]. In fact, nitrogen monoxide (NO) has a high affinity for iron and can form dinitrosyl-dithiolato-Fe complexes (DNICs), interfering with the normal function of iron-containing enzymes involved in DNA synthesis, mitochondrial electron transport chain, and aconitase, among others [102,103,104].

## 9. Proposed Biomarkers of Ferroptotic Cell Death

As it has been mentioned in previous sections, ferroptosis has distinctive characteristics including morphological changes (shrunken mitochondria), the involvement of lipid peroxidation, as well as the expression of key genes indicating the participation of this type of cell death. Among these genes, the increased expression of cbr3, acsl4, and ptgs2 are associated to the presence of ferroptosis. On the other hand, the decreased expression of gpx4 and slc7a11 has been associated with ferroptosis [10]. While a specific biomarker of ferroptosis is not currently available, the understanding of the pathways related to the different types of cell death (including ferroptosis), as well as the potential liver diseases where iron and oxidative stress play a pivotal role, will help to identify more specific biomarkers of ferroptosis, and finally, to associate them with specific clinical outcomes. 

## 10. Ferroptosis and Liver Disease

Iron-overload disorders include primary and secondary iron overload that can be further categorized as defects in the hepcidin–ferroportin axis, impairing iron transport and causing ineffective erythropoiesis [60]. The most representative disease related to iron overload is hemochromatosis. More than 100 years ago, the association between iron deposits and liver damage was described as hemochromatosis, and subsequently, the mechanisms involved in chronic inflammation, genetics, and cellular damage have been elucidated. Iron-mediated cellular injury is the basis of iron-overload disorders, resulting in organ damage including in the brain, heart, pancreas, and liver [105].

Iron overload in liver diseases arises from two sources: (i) increased intestinal absorption following iron-mediated cellular injury (described above), and (ii) increased iron burden present in some diseases such as β-thalassemia, where frequent repeated blood transfusions are required. Regardless of the cause of iron overload, uncontrolled free iron exerts significant oxidative damage in the liver, contributing to the progression of disease and the development of complications such as HCC [106]. Indeed, there is growing evidence supporting the role of iron as a mediator of liver injury and disease beyond well recognized iron-overload disorders such as hemochromatosis and β-thalassemia [16,17].

### 10.1. Ferroptosis in Metabolic Liver Diseases: Hereditary Hemochromatosis | Non-Alcoholic Fatty Liver Disease (NAFLD)

#### 10.1.1. Hemochromatosis (HH)

In hereditary hemochromatosis (HH), the role of iron in initiating and perpetuating liver damage with further liver fibrosis and HCC has been well described [107]. In particular, HH is caused by genetic mutations, such as in genes encoding hemochromatosis protein (HFE) or SLC40A1 (Ferroptin-1), whose proteins are involved in limiting iron absorption [108]. Moreover, aggressive therapies aimed to decrease iron content through phlebotomy and iron chelators have proven useful in these patients, showing an improvement in several outcomes, including portal hypertension [109]. 

A striking feature in patients with HH is the development of diabetes, in addition to liver damage. The proposed causes of diabetes in HH include decreased pancreatic β-cell function secondary to apoptosis and increased ROS, decreased insulin secretory capacity, and decreased sensitivity to glucose-induced insulin secretion [110]. Finally, an improvement in metabolic outcomes, including higher insulin sensitivity, has been documented after bloodletting in some studies [111,112].

#### 10.1.2. Non-Alcoholic Fatty Liver Disease (NAFLD)

The role of altered iron metabolism in NAFLD has been extensively studied in recent years. The association between iron and fatty liver comes from the link between iron and the development of metabolic syndrome features, [113] including diabetes in diseases such as HH and iron overload secondary to multiple transfusions (referred as the dysmetabolic iron overload syndrome, or DIOS) [114,115]. In these patients, insulin resistance (IR) correlates with the increase in serum ferritin, and half of them display some degree of NAFLD [116,117]. Interestingly, a recent study has shown that increased serum hepcidin levels correlates with liver iron content in NAFLD patients with DIOS [118]. 

Furthermore, augmented levels of serum ferritin in patients with NASH not only are linked to disease severity, including hepatic fibrosis and inflammation, but they also correlate with hepatic iron deposits [119,120,121,122,123]. Recent studies have shown an increased duodenal iron absorption in these patients after oral challenge with iron through the upregulation of DMT1 mRNA in duodenal tissue and the further activation of IRP1 (iron regulatory protein 1) [124]. Although these data suggest iron-lowering therapies as a therapeutic approach in NASH, including phlebotomy, they have been shown to be useful only in particular patients with NASH, with evidence of no benefit in a further randomized clinical trial [125,126,127]. 

### 10.2. Ferroptosis in Alcoholic Liver Disease (ALD)

Active alcohol consumption has been associated with hepatic iron overload mediated by different mechanisms including low hepcidin levels that, in turn, can increase the duodenal iron transport via increased duodenal DMT1 and ferroportin expression [128,129,130,131]. In addition to these mechanisms, a synergistic effect of alcohol and iron increasing liver fibrogenesis and oxidative stress has been proposed. In fact, feeding rodents with carbonyl iron together with a liquid ethanol diet causes elevated serum transaminases levels, steatosis, as well as inflammation and fibrotic markers. This evidence suggests that dietary iron supplementation to an ethanol diet exacerbates hepatocellular damage and promotes liver fibrogenesis which, at least in some experimental cases, leads to cirrhosis and HCC [132,133,134,135,136]. It is of note that the high hepatic iron content in alcohol-related cirrhosis patients has been associated with adverse outcomes, including mortality [137]. 

### 10.3. Ferroptosis and Viral Hepatitis

Chronic hepatitis C virus (HCV) infection can induce iron overload through different mechanisms including (as with ALD) the suppression of hepatic hepcidin, which is caused by HCV-induced oxidative stress, leading to upregulation of duodenal ferroportin-1 [138,139]. Iron overload in HCV has been linked to progressive liver damage, with poorer outcomes being used as a surrogate marker for the severity of the disease in this population [140,141]. Interestingly, transferrin receptor protein 1 (TfR1) has been described in HCV entry facilitating virion internalization, as well as in HCV-driven changes in iron metabolism hepatocyte–KC cross-talk that promotes enhanced viral replication and translation [142,143,144].

Furthermore, changes in iron kinetics are observed during treatment with PEG-IFN/ribavirin, observing an acute increase in serum hepcidin levels 24 h after treatment with a further increase in iron and ferritin levels [145,146,147]. These dynamic changes could be involved in the viral kinetics associated with treatment, and they might be part of a reactive response of macrophages toward IFN.

### 10.4. Ferroptosis and Drug-Induced Liver Injury (DILI)

Drug-induced liver injury (DILI) is the predominant cause of acute liver failure (ALF) in Europe and the USA, with acetaminophen (APAP; paracetamol) as the model hepatotoxin [148]. Although the pathophysiological mechanisms driving APAP toxicity have been extensively studied, there are also characteristics that implicate the participation of ferroptosis. The main feature of APAP toxicity is the formation of N-acetyl-p-benzoquinone imine (NAPQI), which is a highly reactive and toxic APAP metabolite. NAPQI is normally detoxified by glutathione (GSH); however, APAP overdose results in excess NAPQI formation and the subsequent depletion of the GSH antioxidant [149]. In addition, some studies have shown that LPO is essential in the mechanism of APAP-induced cell death, whilst both Vitamin E and iron chelators have been used to ameliorate this damage in susceptible animals (i.e., vitamin E deficiency) [150]. In a recent study, challenge with ferrostatin-1, a specific ferroptosis inhibitor, to primary mouse hepatocytes treated with APAP led to increased cell viability. Since ferrostatin-1 was found to have no influence on CYP2E1 or cellular GSH content, it can be inferred that its protective effect on APAP-induced cell death is independent from interfering with APAP metabolism to NAPQI [151]. Thus, these findings support the potential involvement of this type of cell death in DILI.

Antioxidants such as vitamin E and N-acetyl cysteine (NAC) have been successfully used in non-APAP DILI, including studies of hepatotoxic effects of sulfasalazine and anti-tuberculosis drugs among others [152], which underscores the important role of ROS in DILI. Furthermore, mutations in the GST gene leading to deficiency in glutathione S-transferase activity increase the risk of hepatotoxicity upon treatment with antituberculosis drugs [153]. 

Taken together, these findings suggest ferroptosis as a plausible mechanism involved in some types of DILI. This mode of cell death may be a transient or a sequential phenomenon that follows the initial damage that progresses to glutathione depletion, cell damage, and the release of intracellular components, including iron [154]. 

### 10.5. Ferroptosis and Hepatocellular Carcinoma (HCC)

Other diseases have been associated with iron overload, the consequent activation of HIF-α, and the further decrease of hepcidin expression, including HCC, with increased risk in specific populations, mainly in those carrying the mutation C282Y in the HFE gene (homeostatic iron regulator), which leads to higher hepatic iron deposition and serum ferritin [155,156,157]. 

In several types of cancer, ferroptosis has been proposed as a strong inhibitor of tumor growth, and in other types of cancer, it has enhanced the sensitivity to chemotherapeutic drugs [158]. However, the exact role of ferroptosis in HCC is still not fully elucidated. Several studies have shown that the cytotoxic effect of sorafenib (a multikinase inhibitor used for HCC treatment) in HCC derived-cell lines could be explained by the induction of oxidative stress and iron-dependent cell death that resembles ferroptosis, but not other types of cell death such as apoptosis or autophagy. Moreover, these effects were completely blocked by using ferroptosis inhibitors, including ferrostatin-1, suggesting a key role of ferroptosis in the mechanism of pharmacologically-induced cell death induction in HCC [159,160]. Furthermore, it is known that many HCC cells lose retinoblastoma (Rb) protein function. A recent study showed that HCC cells with decreased levels of Rb displayed a higher rate of cell death after sorafenib exposure. This effect had *in vivo* implications demonstrated by the fact that nude mice receiving tumor xenografts from HCC with low Rb expression had a high level of tumor regression after sorafenib treatment. This demonstrates that an Rb-negative status in HCC could be in fact be responsible for the effectiveness of Sorafenib via ferroptosis [161,162].

It has also been demonstrated that Nrf2 has a protective role in HCC against ferroptosis. Specifically, when HCC cell lines are exposed to erastin or sorafenib, activation of the p62–Keap1–NRF2 pathway prevents Nrf2 degradation, promoting p62 nuclear accumulation and leading to the activation of several factors that inhibit ferroptosis [163]. 

## 11. Experimental Models of Iron Overload and Liver Damage

Different animal models have been developed to elucidate the role of iron in liver disease. Studies in animals include the exogenous administration of iron and/or genetic modifications altering and promoting iron overload [164,165,166].

A comprehensive review showing some knockout models in mice for the study of iron overload, including *Hfe*^-/-^ and iron regulatory protein (*Ipr2*)^-/-^ mice, has been published elsewhere [167]. These models have helped define the role of the different receptors and molecules on iron overload and their importance in liver disease, as well as the mechanisms linked to cell damage elicited by iron. Some approaches that are aimed at selectively deleting or down-regulating iron-related genes in specific tissues can be accomplished through the Cre-loxP system or siRNA knockdown. These techniques have shown the relevance of specific components of iron metabolism in a particular type of cell. 

Genetically modified mice include models resembling hemochromatosis, disruption in mitochondrial iron metabolism, alterations in iron trafficking through the body, and signaling to hepcidin. In particular, hepcidin knockout mice displayed significantly increased iron absorption and overload that led to elevated liver enzymes, mild hepatic inflammation, and moderate liver fibrosis after feeding them with an iron-rich diet [168,169].

## 12. Clinical Implications for the Study of Ferroptosis

For clinicians, it is necessary to understand the fine balance between iron deficiency and iron overload, especially in the context of chronic liver disease, where chronic infection and/or inflammation could be exacerbated by iron supplementation. This needs to be counterbalanced with the fact that iron-deficiency anemia is a frequent finding in chronic liver diseases, which has been associated with adverse outcomes and exacerbation of some complications of cirrhosis such as hepatic encephalopathy and decreased quality of life [170,171]. 

In patients with cirrhosis, hemoglobin levels inversely correlate with hepatic venous pressure gradient, and the presence of anemia is associated with a worsened hyperdynamic circulation in portal hypertension [172,173]. Increased inflammation and the further production of IL-6 and IL-1(R) increases hepcidin transcription, leading to hypoferremia and finally to anemia [61].

The recognition of ferroptosis as a mechanism of liver disease could help to better understand the complex relationship between specific components occurring at a particular moment of liver cell damage and the elicited response of the tissue as a whole. In addition, new markers of ferroptotic hepatic cell death and the development of ferroptosis inhibitors (described below) could help to impede progression and/or lead to the reversion of liver damage triggered by different stressors.

## 13. Pharmacological Modulation of Ferroptosis

Although several biomarkers have been proposed as indicators of ferroptosis, at present, there are no specific and reliable markers of this mode of cell death. Most studies concerning ferroptosis are based on the different changes elicited upon erastin administration (described above).

Modulators of ferroptosis can be classified as inducers and inhibitors (Table 2) depending on their effect at some stage of the pathway. Inducers can be further classified as type 1 (inhibitors of the Xc^-^ system) or type 2 (direct inhibitors of gpx4). Although specific compounds modulating this pathway have been recently developed, repurposed drugs targeting iron overload-related diseases such as hemochromatosis and secondary iron overload (e.g., multiple transfusions) have been proven to be useful in attenuating damage triggered by ferroptosis. Some of these drugs include deferoxamine, deferasirox, and deferiprone, through which their effect as iron chelators could help to prevent iron-driven damage. 

Other drugs that can modulate ferroptosis include sulfasalazine, sorafenib, and some lipophilic antioxidants such as Vitamin E. Their role as ferroptosis modulators has been recently recognized, which could explain to some extent their observed clinical effects. Recently, a class of drugs specifically designed to target ferroptosis has been developed; among these compounds, ferrostatin-1 and liproxstatin-1 are the best categorized. The characteristics of these compounds are shown in Table 2.

## 14. Conclusions and Future Perspectives

The characterization of the specific mechanisms of hepatocyte cell death is important in order to understand the pathophysiological pathways of liver damage inherent to the etiology of the liver disease [6,9]. It is also relevant to develop strategies aimed at halting progression of the damage, and new targeted therapies that allow greater clinical efficacy with minimal side effects. Emerging studies show that ferroptosis is a novel and determinant type of regulated cell death involving the activation of signal transduction pathways that affect diverse hepatic cell populations in different experimental models of liver disease [34,159]. Currently, there are some challenges in ferroptosis. One of them is the lack of a specific marker suitable for use both in animal studies, as well as in the clinical setting. As the research in the field progresses and our understanding of the mechanisms associated to this type of cell death increases, it will be possible to better (and easily) characterize the presence of ferroptosis. On the other hand, the role of lipidomics and the interaction with iron needs to be thoroughly studied in liver diseases.

Finally, delimiting more precisely the presence of ferroptosis will open the possibility of new therapeutic options, as well as the development of specific biomarkers in liver diseases, and better understanding the complex series of events following initiation of inflammation leading to fibrosis, cirrhosis, and end-stage carcinogenesis.

## Figures and Tables

**Table 1:** Characteristics of the different types of cell death.

	Apoptosis [9,20,21]	Necrosis/Necroptosis [6,9,22,23,24]	Pyroptosis [25,26,27]	Autophagic Cell Death [28,29,30,31,32]	Ferroptosis [10,33,34]
Morphological changes	Shrunken cells, membrane blebbing, nuclear condensation and fragmentation	Oncosis, swelling of the organelles and practically no change in the nuclei until later stages when chromatin condensation is observed	Plasma membrane rupture, pyroptotic body formation, and cell flattening	Formation of autophagosomes	Shrunken, electron-dense mitochondria and rupture of the outer mitochondrial membrane
Triggering stimuli	DNA damage and reactive oxygen species (ROS) overload or endoplasmic reticulum (ER) stress (intrinsic), extracellular microenvironment alterations and mediated by death receptors (DRs) (extrinsic)	Physicochemical stress in the cells, detected by TNFR1, Fas, or TLR-3/4	Extracellular stimuli (e.g., TNF, IFNγ and TLR ligands) and different intracellular pathogens	Metabolic stressors	Glutamate, pharmacological induction (erastin, sulfasalazine, sorafenib)
Main components in the pathway	Caspases: initiation (caspase 2, 8, 9 and 10) and execution (caspase 3, 6 and 7)	(RIPK1), RIPK3, and mixed lineage kinase domain-like (MLKL)	Inflammasomes, caspase 1, IL-1*ß*, and IL-18	ATGs proteins, acid hydrolases	Iron, GPX4, ACLS4, SLC7A11, PTGS2

**Table 2:** Proposed mechanism of action and common uses of the different ferroptosis modulators.

Compound	Molecular Target/Mechanism of Action	Common Use, Notes
**INDUCER**		
Erastin [174,175,176]	Inhibits Xc^-^ system (irreversibly)	Ferroptosis inducer in research
RSL3 [177,178]	Inactivates gpx4	Ferroptosis inducer in research
Glutamate [179]	Competitive inhibition of the Xc^-^ system	High concentrations inhibit the function of the antiporter, lowering the intracellular levels of GSH and therefore increasing oxidative damage.
Sulfasalazine [179,180]	Inhibits Xc^-^ system	Patients with inflammatory bowel disease and arthropathies. Used in research in different types of cancer (v.gr. lymphoma, CNS tumors)
Sorafenib [160]	Multikinase inhibitor/inhibit Xc- system	Used mainly as a therapy in patients with advanced hepatocellular carcinoma
**INHIBITOR**		
Ferrostatin-1 [181]	Interferes with ROS accumulation from lipid peroxidation	Second- and third-generation ferrostatins are more stable.
Liproxstatin-1 [15]	Interferes with ROS accumulation from lipid peroxidation	Relative potency stronger than Ferr-1. Inhibits FINs (RSL3, erastin).
Zileuton [182]	Inhibits 5-LOX (abrogates cytosolic ROS production)	Available as an oral compound.
DFO [34,174]	Iron chelator	Used in patients with iron overload
Vitamin E and analogs (v.gr. Trolox) [183,184]	Antioxidant/ROS scavenger	Some trials have tested its effect (e.g., age-related macular degeneration, dementia, metabolic diseases, NAFLD) without conclusive results
1,10-phenanthroline [185,186]	Iron chelator	Used as a metal chelator and redox indicator. Mixed with different metals (Cu, Mn, Ag) has antimicrobial activity
deferasirox [187,188]	Iron chelator	Used in patients with iron overload
deferiprone [187,188,189]	Iron chelator	Used in patients with iron overload

RSL3, Ras-selective lethal 3; gpx4, glutathione peroxidase 4; DFO, deferoxamine; CNS, central nervous system; ROS, reactive oxygen species; 5-LOX, 5-lipoxygenase; FINs, ferroptosis-inducing compounds.

## Abbreviations

Acsl4	Acyl-CoA Synthetase Long Chain Family Member 4
APAF1	Apoptotic Protease-Activating Factor-I
APAP	Acetaminophen
AREs	Antioxidant Response Elements
ASH	Alcoholic Steatohepatitis
ATGs	autophagosomes
ATP	Adenosine Triphosphate
Bcl-2	B-cell Lymphoma 2
BH3	Bcl-2 Homologous 3
BMP	Bone Morphogenetic Protein
Cbr3	Carbonyl Reductase 3
CNS	Central Nervous System
COX	Cyclooxygenase
Cul3	Cullin 3
DAMP	Damage-Associated Molecular Pattern
DATP	Deoxyadenosine Triphosphate
D-cytb	Duodenal Cytochrome b
DFO	Deferoxamine
DILI	Drug-Induced Liver Injury
DIOS	Dysmetabolic Iron Overload Syndrome
DMT1	Divalent Metal-Ion Transporter 1
DNA	Deoxyribonucleic Acid
DNICs	Dinitrosyl-Dithiolato-Fe Complexes
DR	Death Receptor
DsRNA	Double Stranded RNA
EpREs	Electrophile Response Elements
ER	Endoplasmic Reticulum
FADD	Fas-Associated Death-Domain
FAS	Fas Cell Surface Death Receptor
Fe3^+^	Ferric Iron
Fe2^+^	Ferrous Iron
FINs	Ferroptosis-Inducing Compounds
FLIP	FLICE-Inhibitory Protein
Fpn1	Ferroportin
GCL	Y-Glutamyl-Cysteine Ligase
GGC	Y-Glutamyl-Cysteine
GPX	Glutathione Peroxidase
Gpx4	Glutathione Peroxidase 4
GSS	Glutathione synthetase
GSDM	Gasdermin
GSH	Glutathione
GST	Glutathione *S*-Transferase
H_2_O_2_	Hydrogen Peroxide
HCC	Hepatocellular Carcinoma
HCV	Hepatitis C Virus Infection
HH	Hereditary Hemochromatosis
HFE	Homeostatic Iron Regulator
HNE	4-Hydroxynonenal
INF	Interferon
IRP1	Iron Regulatory Protein 1
*Ipr2* ^-/-^	Regulatory Protein 2
5-LOX	5-Lipoxygenase
KCs	Kupffer Cells
Keap-1	Kelch-Like Erythroid Cell-Derived Protein 1
LOX	Lipoxygenases
LPO	lipid peroxidation
MDA	Malondialdehyde
MLKL	Mixed Lineage kinase Domain-Like
NADPH	Nicotinamide Adenine Dinucleotide Phosphate
NAFLD	Non-Alcoholic Fatty Liver Disease
NASH	Non-Alcoholic Steatohepatitis
NAPQI	N-Acetyl-P-Benzoquinone Imine
NF-κB	Nuclear Factor Kappa-Light-Chain-Enhancer of Activated B Cells
NLR	Nucleotide-Binding Domain–Like Receptors
NO	Nitrogen Monoxide
Nrf2	NF-E2-Related Factor 2
NTBI	non-transferrin-bound iron
PAMPs	Pathogen-Associated Molecular Patterns
PIR	Pirin
Pro-IL	Pro-Inflammatory Cytokine Interleukin
PRRs	Pattern Recognition Receptors
Ptgs2	Prostaglandin-Endoperoxide Synthase 2
PUFA-PL	Polyunsaturated-Fatty-Acid-Containing Phospholipids
PYHIN	Pyrin and HIN Domain
Rb	Retinoblastoma
RIPK	Receptor-Interacting Serine/Threonine-Protein Kinase
RHIM	RIP Homotypic Interaction Motif
ROS	Reactive Oxygen Species
RNS	Reactive Nitrogen Species
RSL3	Ras-Selective Lethal 3
SiRNA	Small Interfering RNA
SLC3A2	Glutamate/Cystine Antiporter Solute Carrier Family 3 Member 2
SLC7A11	Glutamate/Cystine Antiporter Solute Carrier Family 7 Member 11
sMAF	Heterodimer with Small MAF
TfR1	Transferrin Receptor 1
TLR	Toll Like Receptors
TNF	Tumor Necrosis Factor
TNFR1	TNF Receptor Superfamily Member 1A
TRADD	TNF-R Adopter Protein via Death Domain
TRAIL	TNF-Related Apoptosis-Inducing Ligand,
TRIM	Tripartite Motif
